# Higher triglyceride-glucose index and triglyceride glucose-body mass index protect against sarcopenia in Chinese middle-aged and older non-diabetic women: a cross-sectional study

**DOI:** 10.3389/fpubh.2024.1475330

**Published:** 2025-01-07

**Authors:** Min Li, Ying Liu, Lei Gao, Yongli Zheng, Luyao Chen, Yan Wang, Wei Zhang

**Affiliations:** ^1^Department of Endocrinology, Hebei Medical University Third Hospital, Shijiazhuang, Hebei, China; ^2^Department of Radiology, Hebei Medical University Third Hospital, Shijiazhuang, Hebei, China

**Keywords:** sarcopenia, triglyceride-glucose index, triglyceride glucose-body mass index, insulin resistance, middle-aged and older non-diabetic women

## Abstract

**Purpose:**

Sarcopenia, an age-related complication, constitutes a major public health problem given the aging of the population. However, it is frequently overlooked and undertreated in mainstream practice. The study aimed to investigate the correlations between triglyceride-glucose (TyG) index and TyG-body mass index (BMI) and sarcopenia in non-diabetic middle-aged and older women and whether they would be helpful indicators of sarcopenia.

**Patients and methods:**

This cross-sectional study was conducted in the Department of Endocrinology, Hebei Medical University Third Hospital. This study prospectively enrolled 460 non-diabetic postmenopausal women aged ≥50 years.

**Results:**

As TyG and TyG-BMI increased, the prevalence of sarcopenia decreased. In multivariate analysis, the TyG index and TyG-BMI index were inversely associated with sarcopenia (OR: 0.492; 95% CI: 0.256–0.944 and OR: 0.948; 95% CI: 0.934–0.962). Women in the fourth TyG-BMI quartiles showed decreased OR of 0.009 (95% CI: 0.001–0.072) for sarcopenia with respect to first quartiles after adjusting confounding factors. The area under the curve (AUC) for TyG index in the diagnosis of sarcopenia was 0.598 (95% CI: 0.529–0.666), while the AUC for TyG-BMI index was 0.858 (95% CI: 0.816–0.899).

**Conclusion:**

Higher TyG index and TyG-BMI index protected against sarcopenia in non-diabetic middle-aged and older females. Moreover, the TyG-BMI index was a reliable and cost-efficient biomarker to predict sarcopenia.

## Introduction

Sarcopenia is recognized as an important nutritional problem accompanying age-related loss of muscle mass and function that reduces mobility, diminishes quality of life, and can lead to death (1). Estrogen contributes to skeletal muscle health by promoting muscle stem cell proliferation, differentiation, and regeneration (2). Owing to aging and estrogen deficiency, sarcopenia is found to be more prevalent in postmenopausal middle-aged and older women (2). In a population of postmenopausal women, the prevalence of sarcopenia ranges from 10 to 40% (3), undoubtedly increasing the health burden and socioeconomic costs. Sarcopenia has been overlooked in mainstream practice, due to the complexity of determining what variables to measure. Hence, it is critically significant to detect postmenopausal individuals with sarcopenia in advance by a reliable, inexpensive and convenient marker, thus implementing early intervention strategies to lower risk of sarcopenia.

The sarcopenia phenotype has many contributing causes beyond aging. Previous studies reported that insulin resistance (IR) may be contribute to the decline in muscle mass (4), progressively giving rise to sarcopenia. Skeletal muscle is a primary organ for insulin disposal. Insulin plays a major role in boosting muscle protein gain and muscle growth through increasing muscle protein synthesis coupled with inhibiting proteolysis (5). Due to a defect in the insulin signal transduction pathway, the anabolic action of insulin is afflicted, which could result in skeletal muscle loss (5). Moreover, development of IR may induce mitochondrial alterations leading to a reduction in energy production required for muscle contraction (5). Menopause is a natural event for women during their lifespan caused by the cessation of spontaneous menses or ovariectomy with estrogen decreasing and androgen increasing in circulation (6). Several researches have revealed that endogenous estrogens can protect against IR (7, 8). Lower incidence of IR diminishes severely when women reach the postmenopausal situation (9).

Traditional methods for evaluating IR, including hyperinsulinemic-euglycemic clamp and the homeostasis model assessment−estimated insulin resistance (HOMA-IR), are invasive and unavailable in most developing countries, and they may be interfered with by exogenous insulin levels, which limits their applications in clinical practice. The triglyceride-glucose (TyG) index−which formulated by fasting triglycerides and plasm glucose−can accurately assess IR (10) and has been shown to be superior to the HOMA-IR for the identification of several IR related conditions like type 2 diabetes mellitus (T2DM) (11). Obesity, identified by body mass index (BMI), is another principal factor linked to IR. Recently, a combination of TyG and BMI (TyG-BMI) index has been proposed as a reliable and highly sensitive and specific alternative marker of IR (12). Several studies have reported that higher TyG-BMI index is proportionally related to cardiac and cerebrovascular events in the older or female patients (13) and non-alcoholic fatty liver disease incidents in a healthy population (14).

Considering the relationship between IR and low muscle mass, the elevated TyG index and TyG-BMI index may be risk predictors for sarcopenia. However, recent clinical studies reached contradictory results due to different participants. A research based on Korean populations aged ≥ 40 showed that increased TyG index is associated with the risk of low muscle mass (15). A Chinese investigation reported that TyG index was positively correlated with muscle mass in female subjects with T2DM when TyG index was <9 (16). These outcomes suggested that the degree of IR may have different effects on muscle mass. Besides, T2DM is a disorder of glucose metabolism on the basis of severe IR, which may lead to muscle damage beyond IR itself. Moreover, TyG index and TyG-BMI index are not only IR markers, but also indicators of the body’s nutritional status. Hence, further study on the effect of TyG index and TyG-BMI index on muscle mass in the population without significant IR will be helpful for the prevention and management of sarcopenia in clinical practice. To the best of our knowledge, no relevant studies have investigated the association between the TyG index and TyG-BMI index and sarcopenia in non-diabetic Chinese postmenopausal women. Given the important existing gap in this field and its clinical implications, the purpose of the present study was to investigate whether the TyG index and TyG-BMI index are related to sarcopenia and whether they would be suitable for use as indicators of sarcopenia in Chinese non-diabetic postmenopausal women.

## Methods

### Study design and participants

**Figure 1:**
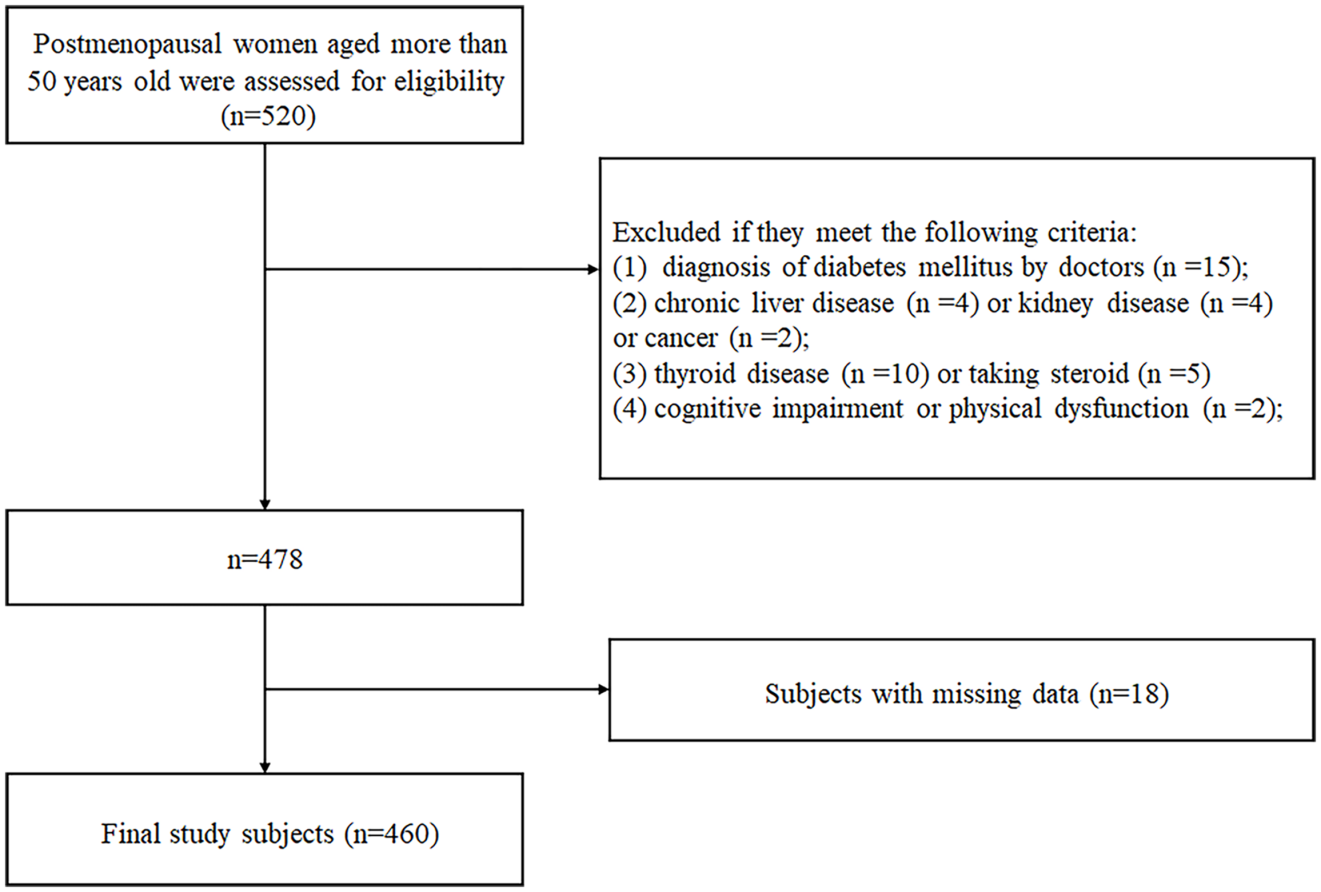
Flow diagram of participant enrollment.

This cross-sectional study was conducted in the Department of Endocrinology, Hebei Medical University Third Hospital from 21th August 2021 to 21th September 2023. The minimum sample size of 378 was calculated using t-tests in G*Power statistical analysis software version 3.1.9.7 considering an effect size of 0.5, alpha error probability of 0.05, power of 0.95, and allocation ratio of N2/N1 of 5, having a sample of 63 sarcopenia and 315 non-sarcopenia. A total of 520 subjects were included based on the following criteria: (1) age more than 50 years old and (2) no menstruation for at least 1 year by self-reporting or at least 6 months after bilateral oophorectomy. We excluded 60 participants who could meet the following criteria: (1) diagnosis of diabetes mellitus by doctors (*n* = 15); (2) chronic liver disease (*n* = 4) or kidney disease (*n* = 4) or cancer (*n* = 2); (3) thyroid disease (*n* = 10) or taking steroid (*n* = 5) that could affect muscle metabolism; (4) cognitive impairment or physical dysfunction (*n* = 2); (5) lack of complete data (*n* = 18) (Figure 1). Finally, there were total 460 postmenopausal women enrolled in the analysis, of which 73 participants were diagnosed with sarcopenia. None of the study participants used hormone therapy or specific supplements for menopause. The study was carried out in accordance with the Declaration of Helsinki and approved by the Ethics Committee of Hebei Medical University Third Hospital (No. ke2023-080-1 and ke2021-045-1). Written informed consents were signed by all individuals prior to participation.

### Data collection

Baseline data including date of birth, menopausal age, height and weight of patients were collected. Total 5 mL fasting blood samples were collected. Levels of fasting plasma glucose (FPG), triglycerides (TG), total cholesterol (TC), low density lipoprotein cholesterol (LDL-C), high density lipoprotein cholesterol (HDL-C), uric acid (UA), creatinine (Cr), alanine aminotransferase (ALT), aspartate aminotransferase (AST) and albumin (ALB) were measured enzymatically by an auto-analyzer (Olympus AU 2700, Japan) in the biochemical laboratory. The TyG index was calculated as ln [triglyceride (mg/dL) × fasting plasma glucose (mg/dL)/2]. BMI was calculated as weight (kg)/height in meters squared (m^2^). The TyG-BMI index was calculated as TyG × BMI.

### Diagnosis of sarcopenia

Appendicular skeletal muscle mass (ASM) was measured using the Dual-energy X-ray absorptiometry (DXA: Lunar IDXAGE, GE, United States). Relative ASM index (RSMI, ASM/height^2^) was calculated. Handgrip strength (HS) was determined by a dynamometer (Jamar, United States). Measurements were taken twice and the mean values were calculated. Six meter walking test was completed by measuring the time taken to walk 6-meter at a normal pace from a moving start, without deceleration, and taking the average result of at least 2 trials as the recorded gait speed (GS). Based on the updated consensus by the Asian Working Group for Sarcopenia 2019 (AWGS, 2019) (17), sarcopenia was defined as (1) low muscle mass (RSMI, women: <5.4 kg/m^2^) plus (2) low muscle strength (HS, women: <18 kg) and/or low physical performance (6 m walking test <1 m/s).

### Statistical analysis

Statistical analysis was performed using SPSS software (version 24) and graphs were performed using GraphPad Prism 8.0 software. The Shapiro–Wilk test was performed to assess the distribution of continuous data. Normally distributed data were presented as mean ± standard deviation (mean ± SD) and compared using independent sample *t*-test. Non-normally distributed data were expressed as median and inter-quartile range (IQR) and compared using Mann–Whitney *U* tests to check the differences between groups. Categorical variables were expressed as numbers and percentages and compared using the chi-square test. The participants were divided into four groups based on their TyG and TyG-BMI quartiles in order to assess the independent impact factors of sarcopenia. Univariate and adjusted multivariate logistic regression analyses were performed to investigate the associations between the TyG index and TyG-BMI index and the risk of sarcopenia according to their quartiles, respectively. Receiver operating characteristic (ROC) curve analysis was done to evaluate the diagnostic value of TyG index and TyG-BMI index for sarcopenia. The best cut-off values were calculated using the Youden index, which were calculated according to the corresponding sensitivity, specificity for each index. A two-sided *p*-value <0.05 was considered statistically significant.

## Results

### Clinical characteristics of the study participants

Among the 460 participants, 73 individuals had sarcopenia, whereas other participants did not have sarcopenia. Table 1 shows the baseline characteristics of participants and the differences between the two groups. Compared with non-sarcopenia women, women with sarcopenia were significantly older, had significantly lower level of BMI, RSMI, HS, GS, ALB, UA, FPG, ALT, and higher level of HDL-C (all *p* < 0.05). Meanwhile, there were no differences in other biochemical parameters including Cr, TC, TG, LDL-C and AST between the two groups (all *p* > 0.05). Compared with women without sarcopenia, both TyG index and TyG-BMI index were significantly decreased in women with sarcopenia (all *p* < 0.05).

**Table 1:** Characteristics of the study participants and differences between women with sarcopenia and non-sarcopenia.

	All patients	Non-sarcopenia	Sarcopenia	*P*-value
Number	460	387	73	
Age (year)	64 (58,68)	63 (56,67.5)	68 (65.5,71)	<0.001
BMI (kg/m^2^)	23.95 (21.76,26.00)	24.44 (22.45,26.36)	20.37 (19.42,22.15)	<0.001
RSMI (kg/m^2^)	6.05 (5.63,6.52)	6.18 (5.84,6.61)	5.20 (5.01,5.32)	<0.001
HS (kg)	22.21 ± 6.05	22.68 ± 6.04	19.74 ± 5.48	<0.001
GS (m/s)	1.07 ± 0.22	1.09 ± 0.22	0.98 ± 0.21	<0.001
ALB (g/L)	45.63 (44.01,47.82)	45.96 (44.30,47.97)	44.24 (41.05,44.61)	<0.001
Cr (μmmol/L)	67.47 (59.46,76.92)	66.99 (59.67,76.46)	69.43 (57.83,80.97)	0.451
UA (ummol/L)	325.21 ± 90.17	330.30 ± 89.77	298.62 ± 88.16	0.006
FBG (mmol/L)	5.47 (5.08,6.05)	5.59 (5.15,6.14)	5.15 (4.75,5.48)	<0.001
TG (mmol/L)	1.51 ± 0.99	1.54 ± 1.04	1.34 ± 0.72	0.127
TC (mmol/L)	5.12 ± 1.14	5.12 ± 1.17	5.13 ± 0.95	0.939
LDL-C (mmol/L)	2.92 ± 0.69	2.92 ± 0.70	2.93 ± 0.67	0.891
HDL-C (mmol/L)	1.41 ± 0.29	1.39 ± 0.28	1.49 ± 0.33	0.012
ALT (U/L)	22.98 ± 13.69	23.55 ± 14.22	19.99 ± 9.93	0.042
AST (U/L)	21.45 ± 7.96	21.73 ± 8.35	19.95 ± 5.25	0.079
TyG	8.63 (8.29,8.98)	8.65 (8.30,9.01)	8.48 (8.15,8.78)	0.008
TyG-BMI	206.68 (184.81,228.10)	212.31 (192.39,231.34)	173.30 (161.68,189.18)	<0.001

Data presented as mean ± standard deviation or median (inter-quartile range). BMI, body mass index; RSMI, relative skeletal muscle mass index; HS, handgrip strength; GS, 6-meter gait speed; FPG, fasting plasma glucose; TG, triglycerides; TC, total cholesterol; LDL-C, low density lipoprotein cholesterol; HDL-C, high density lipoprotein cholesterol; UA, uric acid; Cr, creatinine; ALT, alanine aminotransferase; AST, aspartate aminotransferase; ALB, albumin; TyG, triglyceride-glucose; TyG-BMI, triglyceride glucose–body mass index. Statistically significant (*p* < 0.05) (non-sarcopenia vs. sarcopenia).

### Prevalence of sarcopenia according to quartiles of the TyG index and TyG-BMI index

**Figure 2:**
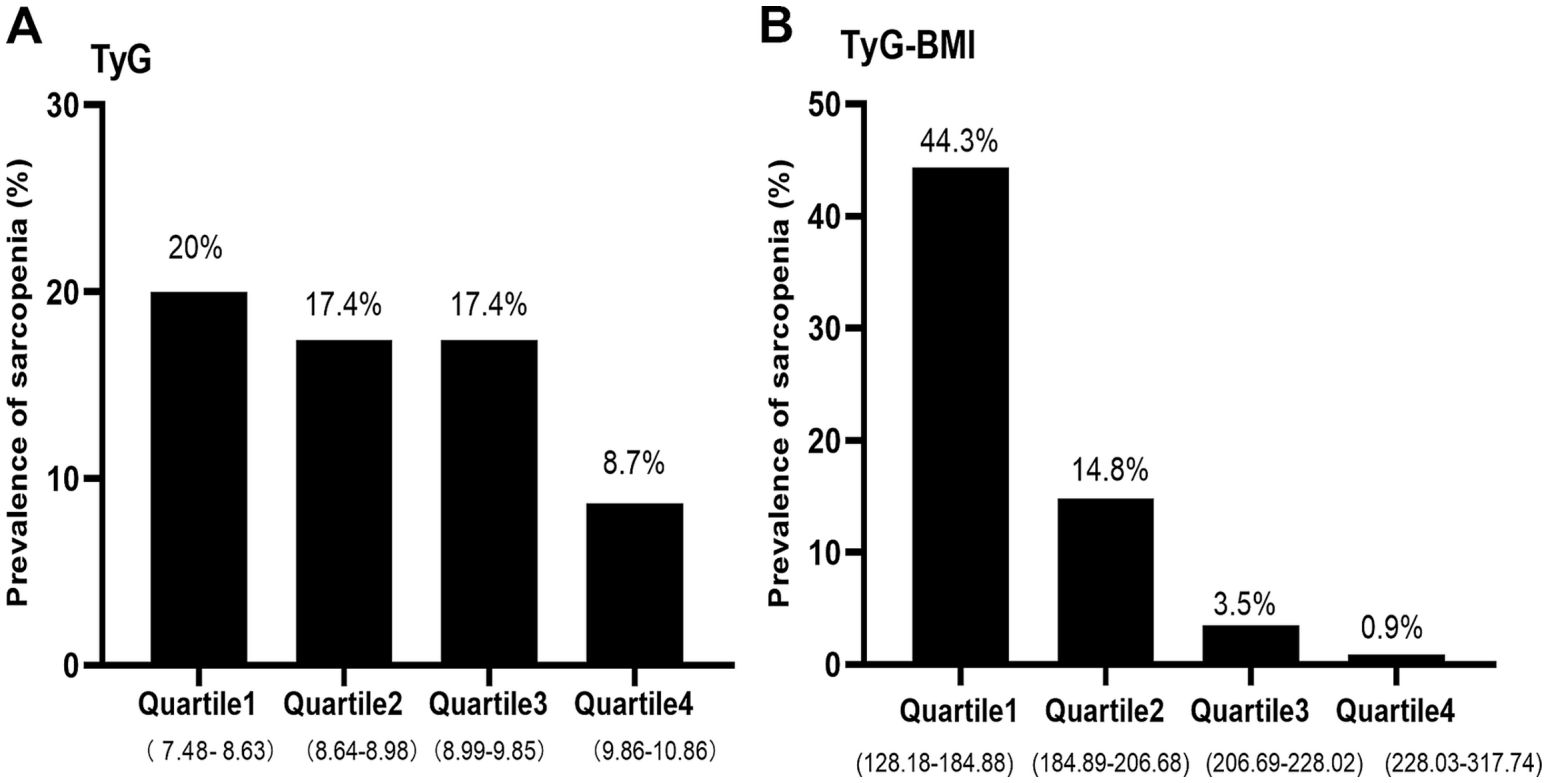
The prevalence of sarcopenia by quartiles of TyG **(A)** and TyG-BMI **(B)** index in non-diabetic, postmenopausal women. TyG, triglyceride-glucose; TyG-BMI, triglyceride glucose–body mass index.

The TyG index and TyG-BMI index were divided into four groups according to quartiles, respectively. As shown in Figure 2, the prevalence of sarcopenia was 20, 17.4, 17.4, and 8.7% in the first, second, third, and fourth quartile of the TyG index, respectively. Meanwhile, the prevalence of sarcopenia was 44.3, 14.8, 3.5, and 0.9% in the first, second, third, and fourth quartile of the TyG-BMI index, respectively. The prevalence of sarcopenia decreased precipitously with increasing quartiles of the TyG-BMI index (*p* < 0.05).

### Associations between TyG index and TyG-BMI index and sarcopenia

Binary logistic regression analysis was done to assess the associations between TyG and TyG-BMI and sarcopenia. As shown in Table 2, in the univariate analysis, the TyG and TyG-BMI index were significantly inversely associated with sarcopenia (odds ratio [OR]: 0.486; 95% confidence interval [CI]: 0.290–0.815; *p* = 0.006) and (OR: 0.948; 95% CI: 0.935–0.961; *p* < 0.001) respectively. In multivariate analysis, the TyG and TyG-BMI index were inversely associated with sarcopenia (OR: 0.492; 95% CI: 0.256–0.944; *p* = 0.033) and (OR: 0.948; 95% CI: 0.934–0.962; *p* < 0.001) respectively after adjusting for confounding variables including age, ALB, Cr, UA, TC, ALT, and AST. As shown in Table 3, in the unadjusted and fully adjusted models, fourth quartile of TyG-BMI showed significantly decreased OR of 0.011 (95% CI: 0.001–0.082; *p* < 0.001) and 0.009 (95% CI: 0.001–0.072, *p* < 0.001) respectively for sarcopenia with respect to its first quartile value. However, there was no significant difference between the quartile 1–4 of TyG index and sarcopenia after rectifying the covariates.

**Table 2:** Binary logistic regression analysis of the independent factors for sarcopenia (TyG and TyG-BMI as continuous variables).

Variable	Model 1		Model 2		Model 3	
	OR (95%CI)	*P*-value	OR (95%CI)	*P*-value	OR (95%CI)	*P*-value
TyG	0.486 (0.290–0.815)	0.006	0.470 (0.269–0.822)	0.008	0.492 (0.256–0.944)	0.033
TyG-BMI	0.948 (0.935–0.961)	<0.001	0.947 (0.934–0.961)	<0.001	0.948 (0.934–0.962)	<0.001

TyG, triglyceride-glucose; TyG-BMI, triglyceride glucose–body mass index; OR, odds ratio; 95% CI, 95% confidence interval; ALB, albumin; Cr, creatinine; UA, uric acid; TC, total cholesterol; ALT, alanine aminotransferase; AST, aspartate aminotransferase. Model 1: not adjusted for any variable; Model 2: adjusted for age; Model 3: adjusted for age, ALB, Cr, UA, TC, ALT, and AST. Statistically significant (*p* < 0.05).

**Table 3:** Binary logistic regression analysis of the independent factors for sarcopenia (TyG and TyG-BMI as categorical variables).

Variable	Model 1		Model 2		Model 3	
	OR (95%CI)	*P*-value	OR (95%CI)	*P*-value	OR (95%CI)	*P*-value
TyG						
Quartile 1	Reference		Reference		Reference	
Quartile 2	0.842 (0.433–1.636)	0.612	0.785 (0.389–1.584)	0.499	0.784 (0.374–1.646)	0.520
Quartile 3	0.842 (0.433–1.636)	0.612	0.721 (0.357–1.455)	0.361	0.711 (0.328–1.540)	0.387
Quartile 4	0.381 (0.172–0.842)	0.017	0.411 (0.180–0.935)	0.034	0.461 (0.181–1.169)	0.103
*p* for trend		0.027		0.039		0.113
TyG-BMI						
Quartile 1	Reference		Reference		Reference	
Quartile 2	0.218 (0.116–0.410)	<0.001	0.197 (0.098–0.394)	<0.001	0.218 (0.105–0.454)	<0.001
Quartile 3	0.045 (0.016–0.131)	<0.001	0.037 (0.012–0.113)	<0.001	0.035 (0.011–0.114)	<0.001
Quartile 4	0.011 (0.001–0.082)	<0.001	0.009 (0.001–0.069)	<0.001	0.009 (0.001–0.072)	<0.001
*p* for trend		<0.001		<0.001		<0.001

TyG, triglyceride-glucose; TyG-BMI, triglyceride glucose–body mass index; OR, odds ratio; 95% CI, 95% confidence interval; ALB, albumin; Cr: creatinine; UA, uric acid; TC, total cholesterol; ALT, alanine aminotransferase; AST, aspartate aminotransferase. Model 1: not adjusted for any variable; Model 2: adjusted for age; Model 3: adjusted for age, ALB, Cr, UA, TC, ALT, and AST. Statistically significant (*p* < 0.05).

### The ROC curves for the TyG index and TyG-BMI index

**Figure 3:**
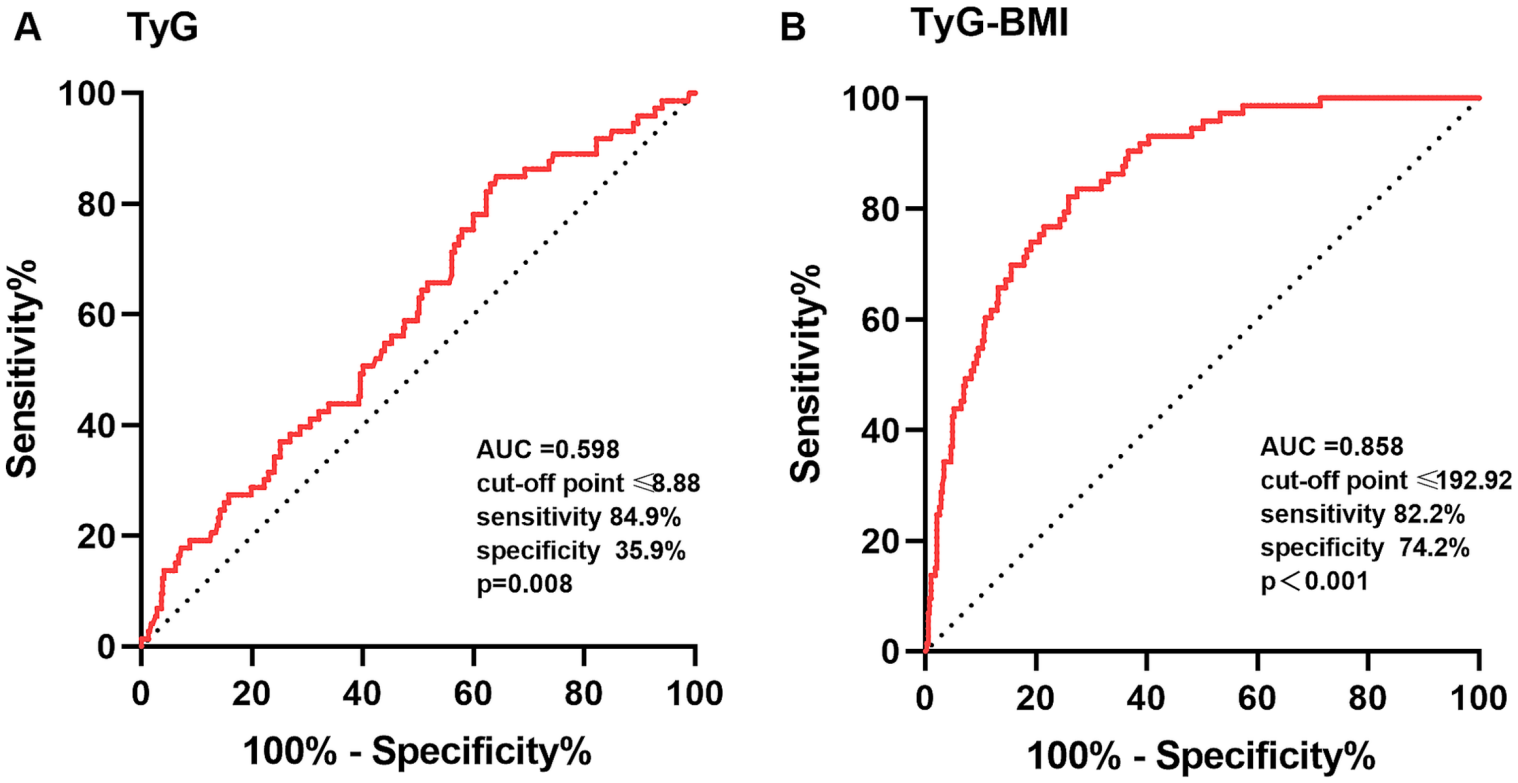
The ROC curves of TyG **(A)** and TyG-BMI **(B)** index for the diagnosis of sarcopenia. TyG, triglyceride-glucose; TyG-BMI, triglyceride glucose–body mass index; ROC, receiver operating characteristic; AUC, area under the curve. The comparison of the AUC was performed by a *p*-value<0.05.

The ROC curves were performed to check the diagnostic value of TyG index and TyG-BMI index for sarcopenia. The area under the curve (AUC) for TyG index in the diagnosis of sarcopenia was 0.598 (95% CI: 0.529–0.666; *p* = 0.008), and at a cut-off point set at 8.88, the sensitivity was 84.9% and specificity was 35.9%. The AUC for TyG-BMI index was 0.858 (95% CI: 0.816–0.899; *p* < 0.001), and at a cut-off point set at 192.92, the sensitivity was 82.2% and specificity was 74.2% (Figure 3).

## Discussion

The current study revealed that sarcopenic women had lower levels of ALB, ALT and UA but higher level of HDL-C, which was consistent with some previous results (18–20). Recent studies have suggested that excessively high HDL-C levels may negatively affect the survival of older adults (21, 22). This negative effect may be stronger than the protective effect of normal HDL-C levels (22, 23). UA, an important antioxidant, was positively associated with muscle mass (24), muscle strength (25) and nutrition score (26). ALT, a transaminase enzyme found in the liver and muscle tissue, has been revealed to be positively associated with muscle mass (27) and negatively with sarcopenia (28) and mortality risk (27). ALB is an indicator of protein metabolic balance and nutrient status. In a meta-analysis, low ALB levels were found to be associated with sarcopenia among older individuals (29).

The BMI is a composite index that reflects body composition, including fat, muscle, and bone tissue. The relationship between BMI and skeletal muscle mass has been intensively studied in different populations but results have been inconsistent. Some studies concluded that higher BMI were significantly related to loss of skeletal muscle and sarcopenia (30, 31) due to systemic inflammation and insulin resistance (32). Quite the reverse, other studies showed that higher BMI protected against sarcopenia in older adults (33, 34) and patients with diabetes (35). Zhang et al. (36) conducted a 4 years of follow-up study in older Chinese populations (females with a mean age of 67 ± 6 in baseline) and found that BMI was positively associated with muscle mass and negatively associated with sarcopenia. In agreement with these studies, our study (females with a mean age of 64 ± 7 years) also showed that BMI is remarkably lower in non-diabetic postmenopausal individuals with sarcopenia with respect to non-sarcopenia group. Overweight may be more beneficial than lower level of normal weight in older-old and vulnerable older people (37). Possible mechanism by which obesity protected muscle mass was as follows. Firstly, skeletal muscle stimulation was increased as a higher level of muscle mass was observed in the obese group (33). Over time, there is less loss of muscle mass owing to the greater load required for exercise (33). Secondly, as hormone levels change with aging, the proportion of fat increases in older individuals (33). Adipose tissue is an essential endocrine organ that regulates hormonal levels like estrogen (34), and abdominal fat in women stores high levels of sex hormones and positively affects skeletal muscle mass (33, 34).

The IR was regarded as a prominent risk factor for sarcopenia or low muscle mass (4, 38). The TyG index is determined as a convenient, cost-effective and reliable indicator for identifying individuals with IR and metabolic syndrome (MetS) in the general population. Higher TyG index has been recently revealed as a potential marker with sarcopenia and low muscle mass in particular individuals (15, 39). TyG-BMI index, which combines serum triglycerides, fasting plasma glucose, and obesity status, is considered more reliable than TyG index for the identification of IR (12). We conducted the study to investigate the association of TyG and TyG-BMI index with muscle mass and sarcopenia in non-diabetic, postmenopausal individuals. Contrary to some previous studies, the present study demonstrated that the TyG index and TyG-BMI index were positively associated with RSMI, and negatively associated with risk of sarcopenia. Similar findings have also been described by Kim et al. (11), in which increased TyG index and BMI were inversely correlated with the incidence of sarcopenia. Nevertheless, the AUCs of TyG index yielded by the ROC curve analysis were only 0.598 for females, which limits its value for early detection of sarcopenia in clinical applications. TyG-BMI index, which incorporates TyG and BMI, appear to be a more useful predictor of sarcopenia than single TyG index for postmenopausal women, with AUC of 0.858.

One explanation was that there was a threshold or saturation effect for TyG index or TyG-BMI index as an indicator of IR. Currently, it was thought that the TyG index ≥8.7 may indicate the presence of IR, regardless of age and sex. Lee et al. reported that non-diabetic and normal weight Korea women with the TyG index ≥8.73 was at a higher risk of metabolic syndrome (40). Similarly, Li et al. (41) reported that the TyG index ≥8.7 was associated with metabolic syndrome in middle-aged and older Chinese, which implied that values above this range were connected with IR. Zhu et al. (42) reported that the TyG index >8.73 and the TyG-BMI index >222.45, respectively, was associated with poor glycemic control in Chinese older individuals with T2DM, which also suggested that it might be related to the development of IR. In our study, the major values of TyG index and TyG-BMI index were less than 8.7 and 222 respectively, which did not meet the threshold for IR. Consist with our results, Hu et al. (16) found that TyG index was positively correlated with SMI in female subjects when the TyG index was <9.

TyG-BMI index—which incorporates fasting blood glucose, serum triglyceride levels and BMI—is also a nutritional indicator within a certain threshold in addition to indicating IR. Nutritional supplementation is required to maintain or improve muscle quality and muscle strength. The present study found that women with sarcopenia showed lower TG than women with non-sarcopenia, however, the difference was not statistically significant. Similar result was found by Yin et al. (19) and Hu et al. (16). Several follow-up studies in Chinese older adults found decreased TG level seem to be associated with increased all-cause mortality risk, which suggests the clinical importance of revisiting the concept of “the lower the better” for the oldest old (23, 43). Lower TG values may be a consequence of poor health status associated with sarcopenia (18). In addition, our investigation also revealed that women with sarcopenia showed dramatically decreased FPG level compared with non-sarcopenia group. Previous study reported that higher glycemic values (glycosylated hemoglobin Alc, HbA1c ≥ 8.5%) in older patients with diabetes were associated with lower muscle mass and muscle quality (44). However, no correlation was found between hyperglycemia and muscle loss in diabetic patients with a mean HbA1c value of 7.0% (45). This discrepancy may be owing to different TG and glycemic concentrations in the study population. Hyperlipidemia and hyperglycemia are widely recognized as risk factors for sarcopenia, nonetheless, the levels of lipid and glucose in our study population are in the normal range. There were no glucotoxic or lipotoxic effects exist but the nutritive supportive effects on muscle synthesis by proper glucose and lipid control.

A relatively higher TyG index is usually accompanied by higher insulin level. Insulin has anabolic effects on muscle mass and protein metabolism in non-insulin resistant men and women (46). In situations where amino acids (AAs) delivery is unchanged, supraphysiological concentrations of insulin are needed to achieve skeletal muscle anabolism (47). Meanwhile, insulin also plays a clear role in reducing muscle protein breakdown independent of AA availability (47). In a cross-sectional study of older Chinese females aged over 50 years, lower-insulin was associated with sarcopenia and risk factors for low muscle mass (19). Sugimoto et al. (48) and Bouchi et al. (49) have also reported that the insulin treatment could improve muscle mass and gait speed and attenuate the progression of sarcopenia in T2DM. All of these results suggested that insulin at the upper limit of the normal range was necessary to maintain muscle mass and function.

In brief, our findings suggested that reasonable weight management should be promoted to guard against muscle damage caused by low body weight in non-diabetic postmenopausal women without apparent IR. Moreover, the risk of sarcopenia caused by hypolipidemia should be considered in patients who require long-term use of lipid-lowering agents. This study provided evidence to support the prevention and treatment of sarcopenia in non-diabetic postmenopausal women.

### Strengths and limitations

There were both strengths and limitations to the current study. To our knowledge, this is the first study to investigate the relationship between TyG index and TyG-BMI index and sarcopenia in Chinese non-diabetic, postmenopausal women. However, this study had some limitations. First, it was a cross-sectional design, which need future longitudinal studies to conclude the cause-and-effect relationship. Second, as a one-center study with a relatively small sample size conducted in Chinese middle-aged and older females, it is unclear whether the findings are applicable to individuals of other ethnicities or in other countries. Therefore, multi-center and multi-ethnic studies with larger sample sizes should be performed to validate conclusions’ reproducibility. Third, some potential covariates, such as dietary intake and supplements, were not examined. The influence of potential unmeasured confounders on the effect of the study indicators on sarcopenia risk needs to be investigated in future studies.

## Conclusion

This cross-sectional study confirmed that there seemed no obvious IR existed in non-diabetic Chinese postmenopausal women. As nutritional indicators, higher TyG index and TyG-BMI index protected against sarcopenia in non-diabetic middle-aged and older females without IR status. Moreover, the TyG-BMI index, with both sensitivity and specificity, was a simple, robust surrogate and cost-efficient biomarker to predict the risk of sarcopenia in non-diabetic Chinese middle-aged and older women. In addition, the current study revealed that sarcopenic individuals had lower values of BMI, ALB, TG, FPG, ALT, and UA, that is to say, individuals with sarcopenia had relative malnutrition. Given this, it may be pertinent to clinically assess nutritional status in older individuals who are at risk for sarcopenia, so as to provide adequate nutritional profile to preserve muscle mass and physical function.

## Data Availability

The raw data supporting the conclusions of this article will be made available by the authors, without undue reservation.
